# Information-Aware Secure Routing in Wireless Sensor Networks

**DOI:** 10.3390/s20010165

**Published:** 2019-12-26

**Authors:** Qiong Shi, Li Qin, Yinghua Ding, Boli Xie, Jiajie Zheng, Lipeng Song

**Affiliations:** 1 Science and Technology on Electronic Test & Measurement Laboratory, North University of China, Taiyuan 030051, Shan’xi, China; shiqiong0641@nuc.edu.cn; 2 School of Instrument and Electronics, North University of China, TaiYuan 030051, Shan’xi, China; 3 School of Computer Science and technology, North University of China, TaiYuan 030051, Shan’xi, China; s1807023@st.nuc.edu.cn (Y.D.); 1607084111@stu.nuc.edu.cn (J.Z.); 4 Department of Mathematics, North University of China, Taiyuan 030051, Shan’xi, China; xieboli@nuc.edu.cn

**Keywords:** secure routing, trust calculation, attack probability, information-aware, global optimization

## Abstract

Secure routing is crucial for wireless sensor networks (WSNs) because they are vulnerable to various attacks. In this paper, we propose a new secure routing protocol for WSNs in the presence of malicious nodes. For each relay node in the route, associated information such as its trust value and status is considered in the protocol. The trust value is defined as the attack probability of the node according to previous packet-forwarding behaviors, and the status is a hybrid metric that combines the residual energy and distance to the sink node. Therefore, the route generated by the protocol is secure against malicious attacks and globally optimal according to the associated information. We used an improved variant of the Dijkstra algorithm to generate the secure route for WSNs in the presence of malicious nodes. Compared with the Reputation-Based Mechanism to Stimulate Cooperation (RBMSC) model in the same simulation environment, the proposed model can maintain a higher delivery ratio, which verifies the effectiveness of the proposed model on the basis of global optimization. Furthermore, compared with the traditional Dijkstra algorithm, the packet loss ratio in the improved Dijkstra algorithm is lower because it can more effectively avoid malicious nodes, thus verifying the effectiveness of the improved algorithm.

## 1. Introduction

Wireless sensor networks (WSNs) have emerged as a promising technology for various application scenarios, such as battlefield monitoring, emergency response, environmental monitoring, and healthcare monitoring. However, WSNs are vulnerable to various threats because they are usually deployed in a hostile or harsh environment [1]. Sensor nodes in WSNs are captured relatively easily by attackers, and then act as malicious nodes that launch various attacks, such as selective forwarding, wormhole, sinkhole, hello flooding, and Sybil [2,3,4]. Malicious nodes drop some or all packets, so some important data cannot reach the sink node if the routing protocol is not immune to such attacks. Even worse, the network might partly or completely fail. The traditional routing mechanisms were designed for data transmission and are thus not applicable to WSNs in the presence of malicious nodes. Furthermore, routing in WSNs is constrained by the energy and computing capacities of sensor nodes. In recent years, the routing security of WSNs has become a hot research topic [5,6,7].

The existing routing protocols for WSNs cannot effectively balance security and energy consumption [8,9,10]. Most of these protocols rely on encryption and trust mechanisms to guarantee security. Although encryption can help WSNs to resist specific types of network attacks, the routing protocols embedded with encryption are vulnerable to other types of network intrusions, which will result in more energy consumption. With more routing paths, we can improve the resilience of these protocols, but the construction and maintenance of extra routes result in more consumption of the energy and storage space of sensor nodes. Trust-based routing protocols can resist various types of attacks, but the calculation of trust values always uses very high memory and consumes substantial energy. The existing routing protocols are typically optimized for energy consumption; thus, a complete route for a pair of nodes is normally generated by augmenting the current path one hop at a time. However, the generated routes are not globally optimal and may fail to work in the presence of malicious attacks. Therefore, it is very hard for existing routing protocols to find a secure route that can balance security and the constraints on the computing and battery capacities of WSNs.

In this paper, we propose a new secure routing protocol for WSNs in the presence of malicious nodes. The protocol finds a secure route by taking into account both the trust value and the status of each relay node in the route. We use attack probability to represent the trust of each sensor node, and the trust value is calculated by the historical communication behaviors of the sensor node and the trust information of its neighbors. With the trust value, we can forecast the future behaviors of the sensor node. Furthermore, we can use the associated information of the sensor nodes to establish a secure route for data transmission between a pair of sensor nodes, which can effectively eliminate interference from malicious nodes. Some sensor nodes may have incomplete information, so we also leverage the indirect trust value from other nodes to enhance the accuracy of the direct trust value.

Our secure routing protocol chooses one sensor node at a time to improve the success rate of data communication without identifying the type of attack. In particular, a sensor node that has more residual energy and is closer to the sink node is selected as the next relay node. Thus, we can reach a trade-off between security and energy consumption for each sensor node. In addition, all secure routes are constructed via the serial–parallel system theory of the classical reliability theory, which guarantees the global optimization of our solution. It is able to not only resist various attacks, but also to choose a route with little interference and no fault in the wireless communication environment.

The contributions of this work can be summarized as follows:

- We propose a globally optimal secure routing method in the presence of malicious nodes. In this method, the security, energy consumption, and load balance of the entire path are comprehensively evaluated by sensing the status and trust of nodes in the network so that the optimal route is found.
- On the basis of the sensed trust value and status, an information-aware secure routing evaluation method is proposed in this paper. A secure route is evaluated by a cost function, which is a comprehensive evaluation of the associated information of the nodes in the path, i.e., the trust value of the path from the source node to the sink node and the status of each node in the path, where the status of a node is a hybrid metric determined by the residual energy of the node and the distance to the sink.
- In the same simulation environment, compared with the Reputation-Based Mechanism to Stimulate Cooperation (RBMSC) model [11], the proposed model can maintain a higher delivery ratio, which verifies the effectiveness of the proposed model on the basis of global optimization. Furthermore, compared with the traditional Dijkstra algorithm, the packet loss ratio in the improved Dijkstra algorithm is lower because it can more effectively avoid malicious nodes, thus verifying the effectiveness of the improved algorithm.

The rest of this paper is organized as follows. In Section 2, we summarize the ideas and methods in the existing literature. Section 3 presents the secure routing model based on information-aware sensor nodes in WSNs. In Section 4, we show the implementation of the proposed secure routing model using an improved Dijkstra algorithm. In Section 5, the effectiveness of our model and algorithm is evaluated by five groups of simulations. Finally, the conclusion and future studies are provided in Section 7.

## 2. Related Work

In recent years, numerous research works have addressed the problem of secure routing in WSNs. The existing methods for secure routing fall into five categories.

1. Identity authentication. Identity authentication is mainly used to determine the sender of information and exchange communication keys between two communication parties. Since nodes in a WSN are relatively easy for attackers to capture, we can use identity authentication mechanisms to verify the captured nodes [12,13,14,15]. Due to the constrained computing and energy capabilities, identity authentication via a symmetric key algorithm is more feasible than the traditional approach based on public key encryption in WSNs [3,16].
2. Message authentication. Message authentication allows the receiver to determine whether a message has been altered in transmission. A code is attached to each message by the sender and verified by the receiver. A possible way to attack a WSN that implements message authentication is to exhaust the limited power of the sensor nodes by sending a large number of fake messages (e.g., flood attacks). However, the attack can be effectively resisted by randomly pre-allocating coupled keys to adjacent nodes or any pair of sensor nodes [17,18].
3. Intrusion detection. Intrusion detection is used to detect and isolate malicious nodes by collecting and analyzing information in WSNs. Intrusion detection mechanisms for traditional networks cannot be directly applied to WSNs because of the limited power, network bandwidth, computing capacity, and storage capacity in WSNs. The authors of [19] proposed an intrusion detection scheme for WSNs based on game theory and an autoregressive model. It is mentioned in [20] that a machine learning anomaly detection system for multicast communication could be constructed.
4. Multipath routing. In this method, we can construct multiple paths for a pair of sensor nodes and only choose one path to transmit data using a certain strategy. Multipath routing can improve the reliability of data transmission and balance the load for the network. In [10,21], multiple paths were established by utilizing the redundancy of wireless sensor nodes to ensure the reliability of data transmission. The authors of [22] presented a multipath routing protocol to ensure network security by building node disjoint paths.
5. Secure routing on trust. Trust is defined as a subjective evaluation, and a node (trustor) is in charge of determining the trust levels of other nodes (trustees) by examining the actions that they perform. The trust values affect the action of the trustor node [8].
  Methods for trust evaluation are usually classified into two categories: Direct and indirect. Direct trust evaluation assesses the past behaviors of participants, whereas indirect trust evaluation assesses the past behaviors of peers in the neighborhood [23,24].
  A trust model is suitable for secure routing in WSNs because it is lightweight. Several trust models have been proposed for secure routing in WSNs, and they are grouped into the following types:
  (a) Bayesian trust model. Through mathematical calculation and data fitting, Bayesian theory can perfectly describe the change law of trust. It utilizes prior probability events to update the latest relevant evidence, and then it predicts subsequent behaviors [25,26,27].
  (b) Game theory trust model. Game theory attempts to explain how to use an opponent’s behavior to make correct decisions about behavioral strategies through mathematical methods. The trust model based on game theory is used to identify and exclude uncooperative selfish nodes from the normal network [28,29,30].
  (c) Fuzzy trust model. The fuzzy trust model relies on a series of fuzzy rules to deal with the uncertainty in trust evaluation. Then, fuzzy theory can be used to evaluate the trust value and select the trusted route [31,32,33].
  (d) Directed and undirected graph model. According to graph theory, a sensor network can be regarded as a graph, sensor nodes can be represented by a set of vertices, and links between sensor nodes are represented by a set of edges between vertices. Trust values are assigned to nodes on the basis of the trust of the adjacent nodes [32,34].
  (e) Weighting trust model. The trust value for each sensor node is determined by the weighted average of the observation values of the sensor node’s past behaviors. With the trust value, we can assess the credibility of the sensor node and improve the capability of intrusion tolerance in WSNs [35,36,37].

In addition, there are some other types of secure routing models. The authors of [38] developed a secure routing protocol named the Trust–Distrust Protocol. Trust (or distrust) of the nodes is graded according to the fitness value obtained by tests and calculations in the protocol. The authors of [39] proposed a trust-based scheme that enforces collaborative behavior in wireless mobile networks by taking peers’ attributes into consideration for an efficient routing scheme. A personalized similarity algorithm for modeling peers’ attributes was introduced as a scaling factor for trust evaluation. The authors of [40] proposed a time-based trust-aware routing protocol for Low-Power and Lossy Networks (SecTrust-RPL). The proposed SecTrust framework is mapped to the Routing Protocol for Low-Power and Lossy Networks (RPL) and provides a new trust-aware RPL protocol.

Although most of the existing routing protocols for WSNs can meet security requirements, they cannot effectively balance security and energy consumption. In this paper, we propose a hybrid model that combines the directed graph model with the weighting trust model, and we refer to the serial–parallel system in classical reliability theory to achieve secure routing construction. In the model, the calculation of trust values is simplified so that it can effectively balance security and energy consumption.

## 3. Model and Methods

In this section, the system model is presented first, and then the methods designed for secure routing in WSNs are discussed in detail.

### 3.1. System Model

We assume that a WSN consists of *n* nodes and adopts a mesh-network structure as its topology. For a WSN G=(V,E,W), V=N∪{sink}, *N* represents the set of sensor nodes, *E* represents the set of edges connecting the nodes, and $W=\{w_{ij}|\left(v_{i},v_{j}\right)$ is an edge of *E*} and is a set of weights associated with edges. *G* has a unique source node (src) and sink node (sink). For any two nodes *s* and *d* of *V*, a transmission path $\langle m_{1},\cdots ,m_{K+1}\rangle$ of length *K* is a *K*-hop path between them, where $m_{1}=s$, $m_{K+1}=d$, and $\langle m_{i},m_{i+1}\rangle$ is an edge of *E*, ∀i ∈{1,⋯,K}. Consequently, a path from the source to the sink in *G* can be written as $l_{s}=\langle src,\cdots ,sink\rangle$. Along with a multi-hop path to the sink, data can be transmitted to the destination. However, in the presence of malicious nodes (i.e., some nodes of *G* are captured by attackers and act as malicious nodes), a *K*-hop path $\langle m_{1},\cdots ,m_{K+1}\rangle$ cannot guarantee successful data transmission between $m_{1}$ and $m_{K+1}$.

### 3.2. Attack Model

In a WSN, a node captured by an attacker becomes a malicious node that initiates an internal attack on other nodes. We consider the selective forwarding attack [41] as an attack model. In a selective forwarding attack, an attacker selectively drops some or all packets so valid data cannot be received. Therefore, the normal collection of data is disrupted. The attack behaviors of such attack nodes are less frequent and disruptive than those of ordinary attack nodes, but they are beyond those of the normal nodes. Therefore, the selective forwarding attack is not easily identified by the detection mechanism.

### 3.3. Attack Behavior Description

The trust value of a node comprises the statistics of historical behaviors and the probability prediction of future behaviors of the node. In our approach, the trust value of a node is evaluated according to its historical communication behaviors. The behavior of a node is divided into normal behavior and abnormal behavior, which is established by evaluating whether the node forwards packets normally.

In this work, sensor nodes can be divided into four categories: Normal nodes, malicious nodes, fault nodes, and dead nodes. According to the attack model in Section 3.2, normal nodes forward the received packets to the next-hop nodes normally, while malicious nodes discard some or all packets deliberately. To simplify the model, we view both fault nodes and dead nodes as malicious nodes because all three types have a significantly increased packet loss ratio.

### 3.4. Behavior Collection

According to the above description, abnormal behavior is mainly reflected by whether the packet is successfully forwarded. Promiscuous monitoring is usually adopted to detect the forwarding packet behaviors of nodes, but this method is not appropriate for WSNs because it leads to large energy consumption by nodes. In our method, packet transmission is confirmed by adopting the two-hop acknowledgment mechanism, which can confirm whether the packet is successfully forwarded by the Acknowledge character (ACK) packet. The working principle is as follows. Node *i* sends a packet to node *j*. If node *j* forwards the packet to node *k*, then node *i* will receive ACK packets from node *j* and node *k*, indicating that node *j* is behaving normally. Otherwise, node *j* is behaving abnormally. It is also presumed that node *j* is behaving abnormally if the ACK packets are not received within the upper bound of time that we set.

### 3.5. Attack Probability

Malicious nodes in the WSN can initiate internal attacks, so we have to find a secure route to guarantee successful data transmission. We use attack probability to evaluate the trust of a route between two nodes. In the next two subsections, we calculate the attack probabilities of a node and a *K*-hop path, respectively.

#### 3.5.1. Attack Probability of the Sensor Node

**Figure 1:**
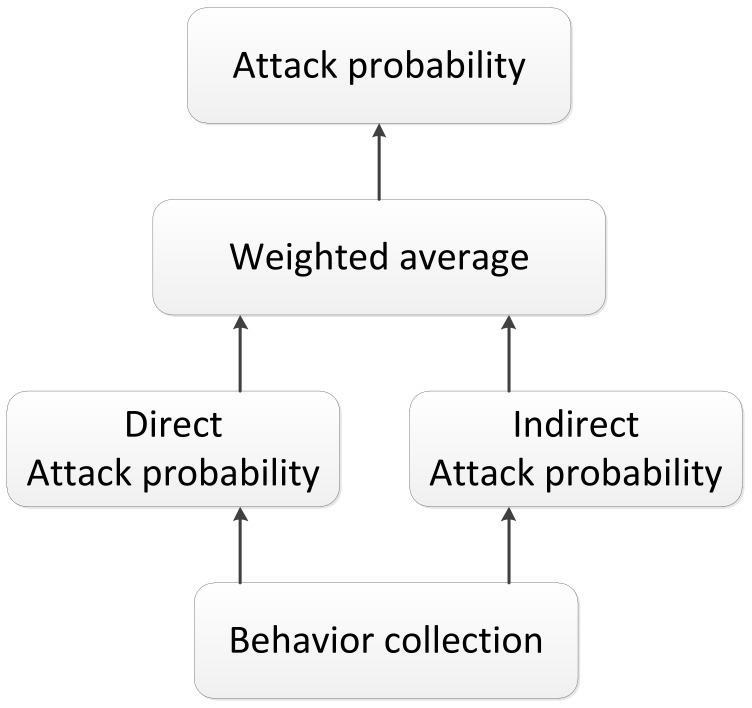
Calculation process of the attack probability.

**Figure 2:**
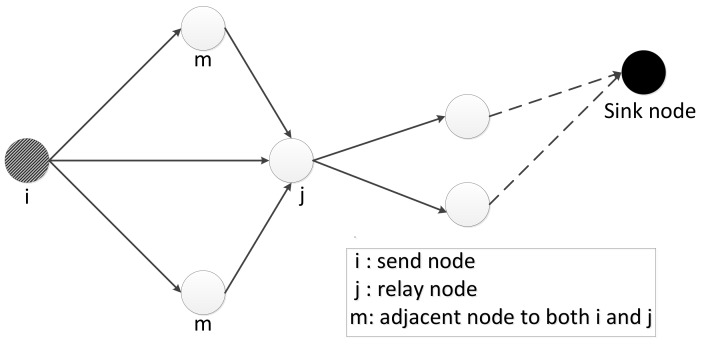
Direct or indirect attack probability.

To find the optimal relay node for a given node *i*, we have to calculate the attack probabilities of its adjacent nodes. Figure 1 and Figure 2 show the calculation method of the attack probability. As shown in Figure 1 and Figure 2, for an adjacent node *j* of *i*, we use the former communication behaviors between them to calculate the attack probability. The attack probability $Attack_{\langle i,j\rangle }$ of *j* to *i* consists of two parts: (1) Direct attack probability $Attack_{d\langle i,j\rangle }$ and (2) indirect attack probability $Attack_{id\langle i,j\rangle }$.

Equation (1) shows the method of calculating the direct attack probability of *j* according to its former communication behaviors with *i*.

(1) $$\begin{array}{cc} Attack_{d\langle i,j\rangle } & =\frac{|P_{ij}-P_{j}|}{P_{ij}}, \end{array}$$

where $P_{ij}$ is the number of packets sent from *i* to *j*, and $P_{j}$ is the number of packets successfully forwarded by node *j* in the past. The direct attack probability is defined as the packet loss ratio of node *j*, i.e., the ratio between the number of lost packets and the number of packets sent to *j* from *i*.

The indirect attack probability, as shown in Equation (2), calculates the attack probability of *j* according to the former communication behaviors with the nodes that are adjacent to both *i* and *j* [11].

(2) $$\begin{array}{cc} Attack_{id\langle i,j\rangle } & =\frac{\sum_{m=1}^{r}\alpha_{m}Attack_{d\langle m,j\rangle }}{\sum_{m=1}^{r}\alpha_{m}},\left(\alpha_{m}=1-Attack_{d\langle i,m\rangle },m\neq i\right), \end{array}$$

where *m* is a node that is adjacent to both *i* and *j*, $\alpha_{m}$ is the trust value of node *m* from *i*, and *r* is the number of nodes that are adjacent to both *i* and *j*. The indirect attack probability of node *j* is determined by the attack probability of *j* to *m*. It is a weighted average of the direct attack probabilities of *j* to all nodes that are adjacent to both *i* and *j*. In this way, even if there are some nodes that deliberately reduce the attack probability of a malicious node by propagating an error value, the attack probability of a malicious node can be calculated by other nodes. Thus, the error has little influence on the overall attack probability, and collusion attacks can be resisted.

Summarizing Equations (1) and (2), we have the attack probability of node *j*, as follows:

(3) $$\begin{array}{c} Attack_{\langle i,j\rangle }=\frac{\sum_{m=1}^{r}\alpha_{m}Attack_{d\langle m,j\rangle }}{\sum_{m=1}^{r}\alpha_{m}},\left(\alpha_{m}=\left\{\begin{array}{cc} 1, & m=i,\\ 1-Attack_{d\langle i,m\rangle }, & m\neq i. \end{array}\right.\right). \end{array}$$

#### 3.5.2. Attack Probability of a K-Hop Path

**Figure 3:**
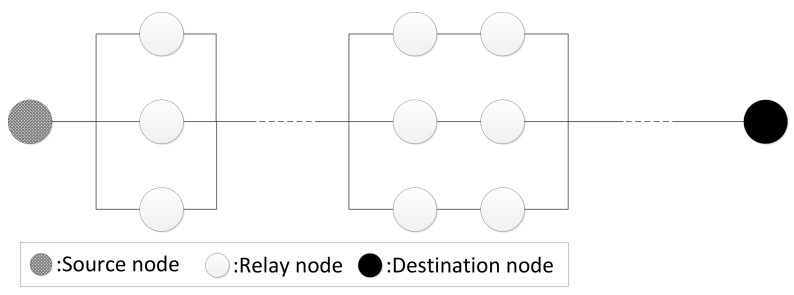
Route in wireless sensor networks (WSNs) based on the serial–parallel system.

As shown in Figure 3, a WSN is very similar to the serial–parallel system in classical reliability theory. A route between two nodes in a WSN can be seen as a serial system, and the WSN can be viewed as a complex parallel system because there is more than one route for each pair of nodes. Data can be transmitted along parallel routes. Data can be successfully transmitted to the sink node along a specific route only if all nodes in the route work normally. Therefore, the nodes in a route can be viewed as a serial system of independent components.

According to the serial system theory, the attack probability of a path ($l=\langle m_{1}\cdots ,m_{K+1}\rangle$) can be calculated as follows:

(4) $$\begin{array}{c} A_{L}=1-\prod_{k=1}^{K}\left(1-Attack_{\langle m_{k},m_{k+1}\rangle }\right), \end{array}$$

where *K* is the number of hops.

### 3.6. Status of Sensor Nodes

In the WSN, the status of each node *j* is associated with two values: (1) The residual energy and (2) its distance to the sink node. The status of each node is used as a heuristic to find the optimal secure route. A node with more residual energy that is closer to the sink node is chosen as the next-hop node. To balance the energy consumption and transmission delay for a sensor node, we define a status value for each node:

(5) $$\begin{array}{c} E_{j}=\frac{\eta e_{\langle j,sink\rangle }}{\eta e_{\langle j,sink\rangle }+e_{rj}}, \end{array}$$

where $e_{\langle j,sink\rangle }$ is the energy required to send a packet from *j* to the sink, and $e_{rj}$ is the residual energy of node *j*. We use a constant η to balance the weight of $e_{\langle j,sink\rangle }$ and $e_{rj}$.

With Equation (5), we can designate a node as the next hop if it has more residual energy and is closer to the sink. We further define the value $E_{L}$ for the entire path:

(6) $$\begin{array}{c} E_{L}=\sum_{k=1}^{K}\left(\frac{\eta e_{\langle m_{k+1},sink\rangle }}{\eta e_{\langle m_{k+1},sink\rangle }+e_{rm_{k+1}}}\times \frac{1}{K}\right). \end{array}$$

The value $E_{L}$ of the entire path is obtained by adding the status values of each node. $E_{L}$ is normalized to ensure that it is always less than 1.

### 3.7. Information-Aware Secure Routing (IASR)

Each node in the WSN is associated with information such as the trust value (attack probability) and status (residual energy and the distance to the sink). We use the attack probability to represent the trust value of a node, and we use the energy consumption required for sending packets from a node to the sink to reflect the distance from the node to the sink.

The secure routing model is information-aware [42]. In this model, each sensor node chooses a path to the sink so as to minimize its cost in the presence of malicious nodes. From the node information collected, the current and future trust value and status of each adjacent node are sensed by the proposed cost function. Therefore, the minimum cost relay node will be found. The cost function for a node accounts for three aspects: (1) The attack probability for the path from itself to the sink, (2) the residual energy of each node in the path, and (3) the distance from each node in the path to the sink.

When a node *n* senses data and transmits it to the sink, it chooses a relay node $s_{n}$ from its adjacent nodes and then forms a *K*-hop transmission path $l_{n}=\langle m_{1},\cdots ,m_{K+1}\rangle$ to the sink in turn. The cost function of node *n* consists of two parts, $c_{n(1)}$ and $c_{n(2)}$, which are calculated with the attack probabilities and status values of nodes in the path, respectively.

(7) $$\begin{array}{cc} c_{n(1)}\; & =A_{L}\\ \; & =1-\prod_{k=1}^{K}\left(1-Attack_{\langle m_{k},m_{k+1}\rangle }\right)\\ \; & =Attack_{\langle n,s_{n}\rangle }-\left(Attack_{\langle n_{,}s_{n}\rangle }-1\right)\left[1-\prod_{k=2}^{K}\left(1-Attack_{\langle m_{k},m_{k+1}\rangle }\right)\right]\\ \; & =Attack_{\langle n,s_{n}\rangle }-\left(Attack_{\langle n,s_{n}\rangle }-1\right)c_{s_{n}\left(1\right)} \end{array}$$

(8) $$\begin{array}{cc} c_{n(2)}\; & =E_{L}\\ \; & =\sum_{k=1}^{K}\left(\frac{\eta e_{\langle m_{k+1},sink\rangle }}{\eta e_{\langle m_{k+1},sink\rangle }+e_{rm_{k+1}}}\times \frac{1}{K}\right)\\ \; & =\left(\frac{\eta e_{\langle s_{n},sink\rangle }}{\eta e_{\langle s_{n},sink\rangle }+e_{rs_{n}}}\times \frac{1}{K}\right)+\sum_{k=2}^{K}\left(\frac{\eta e_{\langle m_{k+1},sink\rangle }}{\eta e_{\langle m_{k+1},sink\rangle }+e_{rm_{k+1}}}\times \frac{1}{K}\right)\\ \; & =\left(\frac{\eta e_{\langle s_{n},sink\rangle }}{\eta e_{\langle s_{n},sink\rangle }+e_{rs_{n}}}\times \frac{1}{K}\right)+c_{s_{n}\left(2\right)} \end{array}$$

The cost function of *n* is

(9) $$\begin{array}{c} c_{n}=c_{n(1)}+c_{n(2)}. \end{array}$$

Equations (7)–(9) show that the cost of a node *n* is determined by the qualities of the current hop in the path and the cost of the adjacent node $s_{n}$ chosen by *n*; thus, global optimization can be ensured.

## 4. Algorithm

First, we distinguish two types of shortest paths in a WSN *G*: (1) The single-source shortest path is the shortest path with a designated source node, and (2) the single-destination shortest path is the shortest path with a designated destination node.

The original problem to be solved in this paper is to find the single-destination shortest path. If we invert the direction of all edges in such a path, we turn it into a single-source shortest path. Therefore, the problem can actually be regarded as the computation of all the shortest paths with a designated sink node in a weighted directed graph, which can be solved by the Dijkstra algorithm [43,44]. In addition, after the problem is converted, the weight is calculated according to the original direction, in order to ensure correctness; that is, the direction of each edge in the graph is not inverted in the actual calculation.

In the Dijkstra algorithm, the cost (weight) of the edge is generally represented by the distance between two nodes. The cost of the path between any two nodes is the sum of the cost of all edges in the path. In contrast, the weight $c_{n}$ in Equation (9) is a hybrid value of the secure cost and the status of the nodes in the path. Therefore, the shortest path in the Dijkstra algorithm is replaced by the minimum cost path in our model.

Algorithm 1 depicts the idea for computing the path with the minimum cost. We first illustrate the notations used in Algorithm 1:

- The sink node is designated as source node $v_{i}$.
- *V* denotes the set of all vertices (V=N∪{sink}). *S* denotes the set of destination nodes that have found the minimum cost path. V−S denotes the set of nodes that have not found the minimum cost path.
- $C_{l(j)}$ denotes the cost of the minimum cost path from node $v_{j}$ to source node $v_{i}$. $tempC_{l(j)}$ denotes the cost of the reconstructed minimum cost path from node $v_{j}$ to source node $v_{i}$.
- $pre_{ij}$ denotes the intermediate nodes in the minimum cost path from node $v_{j}$ to source node $v_{i}$.
- $A_{l(j)}$ represents the total attack probability of the nodes in the entire path from node $v_{j}$ to source node $v_{i}$, $E_{l(j)}$ represents the total status value of the nodes in the entire path from node $v_{j}$ to the source node $v_{i}$, and $C_{l(j)}=A_{l(j)}+E_{l(j)}$.

Initially, *S* only has source node $v_{i}$, i.e., $C_{l(i)}=0$. If source node $v_{i}$ is adjacent to node $v_{j}$, then we set $pre_{ij}=0$ and calculate $C_{l(j)}$. If it is not adjacent to node $v_{i}$, then $C_{l(j)}$ is set to infinity. Then, we add node $v_{k}$ with the least cost in all nodes adjacent to set *S* into set *S*, and the minimum cost path from node $v_{k}$ to source node $v_{i}$ is thereby found. Because node $v_{k}$ joins set *S*, it is necessary to recalculate the cost of the path from each node in set V−S to source node $v_{i}$. We repeat the above steps (by repeatedly selecting the node with the minimum cost path to join set *S*) until set *S* contains all nodes in graph *G*.
**Algorithm 1:** Minimum Cost Path.
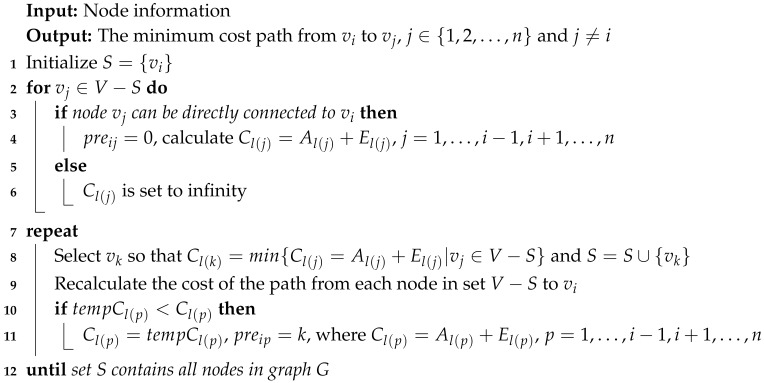


Table 1 compares the improved Dijkstra algorithm and the traditional Dijkstra algorithm.

In regard to the above, the explanations are as follows.

First, it is enough for each sensor node in the network topology to know the adjacent nodes connected to itself. Second, while it is calculating the cost of the adjacent nodes, the sending node requests the adjacent node to send its associated information to itself (the information can be transmitted by means of the message packet); thus, the information of the adjacent node is enough. The associated information includes the trust value of other adjacent nodes, the location and residual energy of the adjacent node, and the cost of the path from the adjacent node to the sink. The secure route in the proposed model is reversely constructed by the Dijkstra algorithm. Therefore, the secure path from the next-hop node to the sink node has been acquired, and the cost value of the path has been calculated in the previous procedure. In the process of secure route selection, it is unnecessary to distribute the information to the whole network, since the local information is enough. In summary, the proposed model can obtain the global optimal path from the source node to the sink node using only the local information obtained.

## 5. Simulations

### 5.1. Scene Setting

In this study, a Virtual Machine (Ubuntu 18.04.2 LTS) and Network Simulator version 3 (NS-3.28) were employed to assess the performance of the proposed model. Simulations were conducted using a simulated WSN, in which 100 nodes were randomly deployed in a 100×100 m square and interconnected with a mesh network. Every node was assigned with the same initial energy and communication radius. Nodes can be divided into four categories: Normal nodes, malicious nodes, fault nodes, and dead nodes. To simplify the model, we view the fault nodes and dead nodes as malicious nodes, so all nodes in the WSN are one of two types.

**Figure 4:**
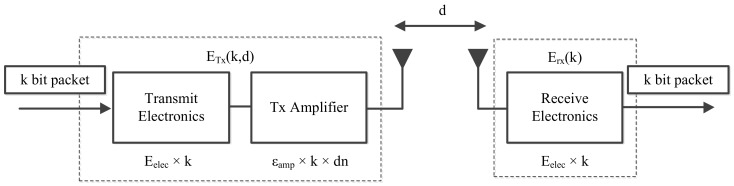
Radio energy dissipation model.

The energy consumption required for the communication between a pair of nodes follows the radio energy dissipation model, as shown in Figure 4. We can see from the figure that a transmitter has two parts—i.e., the radio electronics and the power amplifier—whereas a receiver only has radio electronics. The signal transmitting power in the wireless transmission is exponentially attenuated as the transmission distance increases. This loss can be compensated by the power amplifier. The free space model is employed when the distance between the transmitter and the receiver is less than a threshold $d_{0}$. When the distance is greater than or equal to $d_{0}$, the multipath fading model is adopted. The energy consumed by the sensor node sending the message (*l*-bit) to the receiver (the distance between the transmitter and the receiver is *d*) is expressed as follows [45]:

(10) $$E_{Tx}\left(l,d\right)=E_{Tx-elec}\left(l\right)+E_{Tx-amp}\left(l,d\right)=\left\{\begin{array}{cc} lE_{elec}+l\varepsilon_{fs}d^{2}, & d<d_{0}\\ lE_{elec}+l\varepsilon_{mp}d^{4}, & d\geq d_{0}. \end{array}\right.$$

The energy consumed to receive the message (*l*-bit) is

(11) $$\begin{array}{c} E_{Rx}\left(l\right)=E_{Rx-elec}\left(l\right)=lE_{elec}, \end{array}$$

where $E_{elec}$ is the energy consumption required for electronic devices to accept and transmit 1-bit data. $\varepsilon_{fs}$ and $\varepsilon_{mp}$ represent the free space factor and multipath fading factor, respectively.

### 5.2. Parameter Setting

The parameters in the model are listed in Table 2 and Table 3. The parameters may change in different application scenarios.

### 5.3. Simulation Setup

We ran several simulations to verify the validity of the proposed routing scheme. We calculated the packet loss ratio and delivery ratio in the WSN as follows:

(12) $$\begin{array}{c} P_{LR}=\frac{S-R}{S}, \end{array}$$

(13) $$\begin{array}{c} P_{DR}=\frac{R}{S}. \end{array}$$

In the two equations, *S* is the number of packets sent by the source nodes, and *R* and S−R are the number of packets received by the sink node and the number of lost packets, respectively.

We conducted five groups of simulations to verify the safety and reliability of the proposed routing scheme. In the simulations, we considered two packet-forwarding schemes used by malicious nodes: (1) Random packet loss (RPL), in which a malicious node randomly discards some packets that are supposed to be relayed, and (2) full packet loss (FPL), in which all received packets are dropped. RPL is more confusing and not easily detected (such as a selective forwarding attack). FPL is more harmful but easy to identify. In the G2 simulations, malicious nodes adopted both RPL and FPL schemes, while they adopted RPL in the other simulation groups. In the simulations, under the circumstance of the minimum forwarding threshold τ set, a node with residual energy of less than τ can receive packets but cannot forward them.

Simulations were divided into five groups according to the proportion δ of the malicious nodes in the WSN. The details about each group are as follows:

G1 Simulations: The aim of the first group of simulations was to examine the defense (tolerance) ability of the proposed routing scheme (IASR) against the malicious behavior of sensor nodes. Nodes in the WSN were randomly chosen as malicious, and we examined three levels of the proportion δ of malicious nodes: 20%, 40%, and 60%.

G2 Simulations: In this group of simulations, we compared the performances of the proposed secure routing model (IASR) and the RBMSC model. The packet delivery ratios of the two models were compared under different conditions by varying δ to 10%, 20%, 30%, 40%, 50%, and 60%.

G3 Simulations: The aim of the third group of simulations was to compare the performances of the improved Dijkstra algorithm and the traditional Dijkstra algorithm. We evaluated the ability of the two algorithms to defend against attacks from malicious nodes when δ was set to 20%.

G4 Simulations: This group contained a set of simulations for examining the defense capability comparison of the improved Dijkstra algorithm and the traditional Dijkstra algorithm while δ was dynamically changing over time. Initially, δ was 20%, and then it gradually climbed to 40% and 60% during the simulations. In this case, the historical communication behaviors of the node are unreliable. The information obtained earlier is less reliable. Therefore, this dynamic simulation, which refers to recent historical behaviors, is distinct from the other simulations that reference all historical communication behaviors.

G5 Simulations: This group of simulations examined the effectiveness of the proposed secure routing model (IASR) under a random deployment topology. The simulations were randomly deployed in ten different topologies, and the effectiveness of the model was testified in these topologies.

## 6. Simulation Results

The simulations in each group were repeated 100 times, and the averages were calculated as the final results.

### 6.1. G1 Simulations: Secure Route Construction

**Figure 5:**
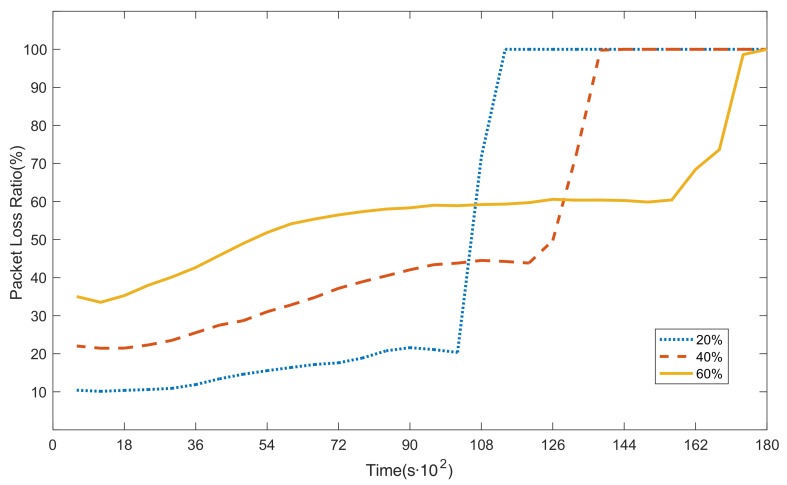
Packet loss ratios under different proportions of malicious nodes (δ = 20%, 40%, and 60%) in information-aware secure routing (IASR).

Figure 5 shows the trends of packet loss ratios under different proportions of malicious nodes in our model. As we can see from the figure, the three lines share similar trends. For δ = 20% (the blue dotted line), the packet loss ratio slowly increases from 10% to 22% in the first 10,200 s, and it suddenly jumps to 100% in the next 1200 s. For δ = 40% (the red dashed line), the packet loss ratio slowly grows from 21% to 45% in the first 12,000 s, and then it suddenly jumps to 100% in the next 1800 s. For δ = 60% (the yellow solid line), the packet loss ratio grows from 33% to 60% in the first 15,600 s, and it suddenly jumps to 100% in the next 2400 s. We observed that the more malicious the nodes, the higher the packet loss ratio in the WSN. The average packet loss ratio exceeds 50% when δ = 60%, and the WSN cannot tolerate intrusions.

**Figure 6:**
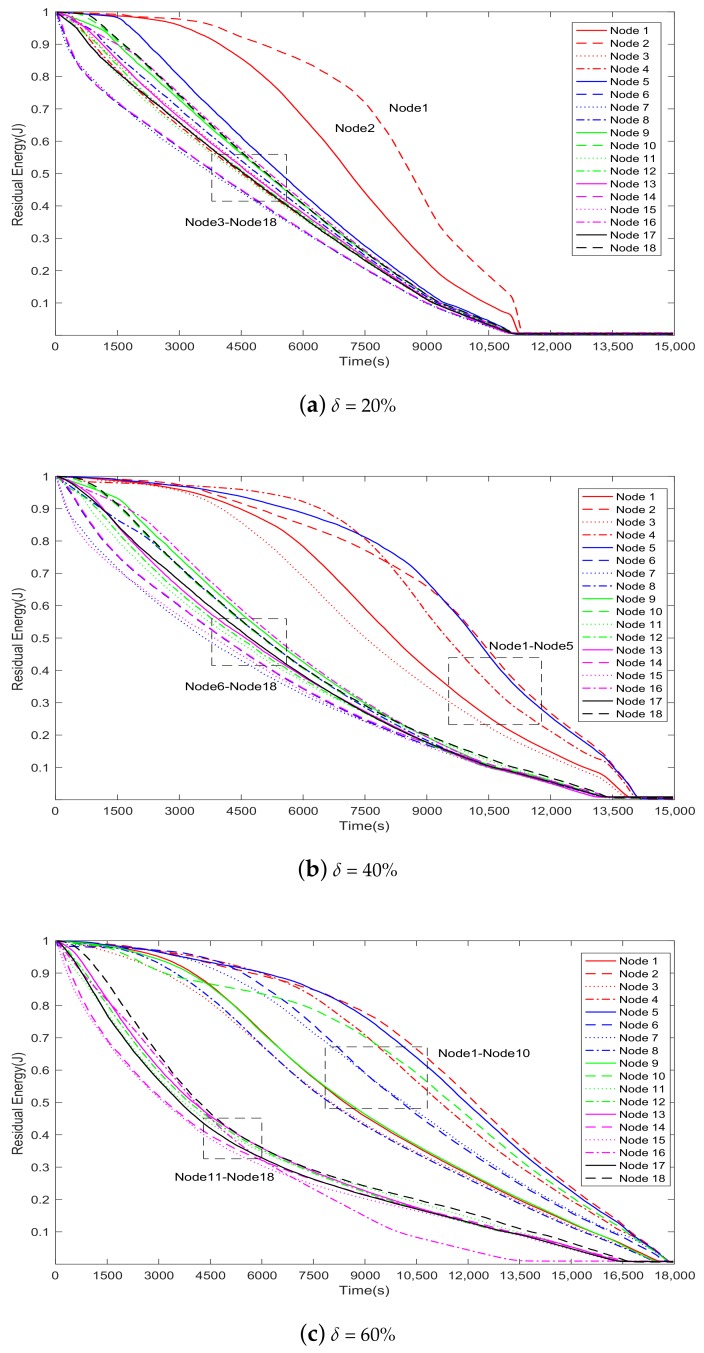
The energy consumption of the 18 nodes near the sink node when the proportion of malicious nodes is 20%, 40%, and 60%.

In Figure 5, for δ = 20% (the blue dotted line), the packet loss ratio suddenly jumps to 100% after 10,200 s. Because the load balance is considered in the model, nodes (especially the neighbor nodes around the sink node) in the network almost run out of energy simultaneously. Therefore, the packet loss ratio rises rapidly to 100% in the next 1200 s. The other two cases (δ = 40% and δ = 60%) are similar to this scenario. The model tries to ensure the load balance of the nodes in WSNs. However, a packet must pass through a limited number of nodes adjacent to the sink node to successfully reach the sink node. These adjacent nodes will run out of energy prematurely and eventually die (as shown in Figure 6). This is the “Hot Spot” phenomenon in WSNs.

Figure 6a–c show the energy consumption of the 18 nodes near the sink node when the proportions of malicious nodes are 20%, 40%, and 60%, respectively.

In Figure 6a (δ = 20%), when the network is run to the 11,130th second, the residual energies of all 18 nodes are less than the minimum forwarding threshold, so they cannot continue to forward packets. Thus, the packet loss ratio rises rapidly to 100%. Note that this moment basically coincides with the moment at which the blue dotted line in Figure 5 rises to 100%.

Similarly, in Figure 6b (δ = 40%) and Figure 6c (δ = 60%), when the network is run to the 13,560th second and the 16,740th second, respectively, the residual energies of all 18 nodes are less than the minimum forwarding threshold. Note that the two moments basically coincide with the moment at which the red dashed line and the yellow solid line in Figure 5 rise to 100%, respectively.

In summary, when the proportion of malicious nodes is 20%, the network packet loss ratio reaches the lowest level, but the packet loss ratio rises to 100% earlier than in the other two cases. In this case, each node (especially the adjacent nodes around the sink node) consumes more energy than the nodes in the other two cases, and the network uses up energy first because it can forward more packets. Therefore, network lifetime in this case is the shortest. Similarly, the packet loss ratio starts to increase earlier and rises more rapidly to 100% in δ = 40% than in δ = 60%.

In addition, Figure 6a–c show that some nodes (nodes 1–2 in Figure 6a, nodes 1–5 in Figure 6b, and nodes 1–10 in Figure 6c)—namely, malicious nodes set up in the simulation—consume less energy than the others. This phenomenon can be explained by two facets. First, malicious nodes are less likely to forward packets and consume a lot of energy, because the proposed model stays away from these nodes to the greatest extent possible. Second, malicious nodes forward packets selectively to economize their energy.

### 6.2. G2 Simulations: Comparative Studies of Two Models

**Figure 7:**
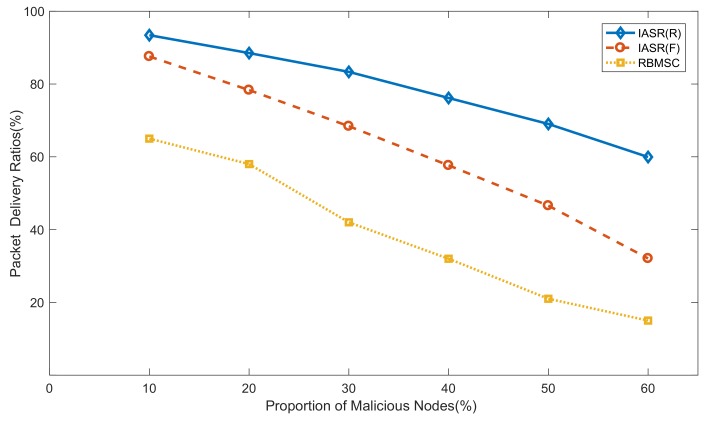
Comparison of packet delivery ratios in two models for different numbers of malicious nodes.

Figure 7 plots the packet delivery ratios of the two models according to the packet-forwarding schemes. The RBMSC model adopted the FPL scheme in the simulation, and it assumes that a malicious node discards all received packets. Objectively, the IASR model verifies both hypotheses that the malicious node processes the packets in the simulation. In Figure 7, the blue solid line, the red dashed line, and the yellow dotted line respectively represent the packet delivery ratios of the IASR model (RPL), the IASR model (FPL), and the RBMSC model (FPL) with different proportions of malicious nodes in WSNs. As shown in Figure 7, the performance of the IASR model is superior to that of the RBMSC model for both assumptions.

As the proportion of malicious nodes in WSNs increases, the packet delivery ratios of both models decrease. Compared with the RBMSC model, the IASR model can maintain a higher delivery ratio. This is because, in the process of path selection, the IASR model not only examines the single-hop optional node, but also evaluates the entire path according to the serial–parallel system of reliability theory to ensure global optimization.

### 6.3. G3 Simulations: Comparative Studies on Algorithms

**Figure 8:**
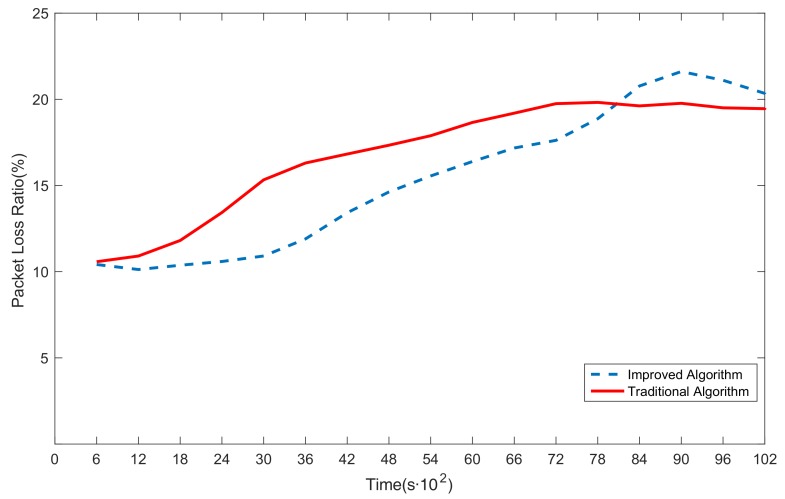
Comparison between the improved algorithm and the traditional algorithm.

Figure 8 shows a comparison between the improved Dijkstra algorithm and the traditional Dijkstra algorithm. As shown in the figure, the packet loss ratio in the improved Dijkstra algorithm (the blue dashed line) is lower than in the traditional Dijkstra algorithm (the red solid line) before 8100 s. The weight in the improved algorithm is not calculated by simply adding the cost of each hop, but by the cost function shown in Equation (9). The cost function is based on the serial–parallel system of reliability theory combined with the characteristics of WSNs, so it is more reasonable and effective than traditional methods. However, the packet loss ratio in the traditional algorithm is lower than the improved algorithm after 8100 s. The reason for this change is that some nodes in the WSN have exhausted their energy because, compared with the traditional algorithm, they deliver more packets in the improved algorithm. Therefore, the key nodes in the late stage of the simulation have exhausted their energy and cannot participate in forwarding packets, resulting in an increase in the packet loss ratio.

**Figure 9:**
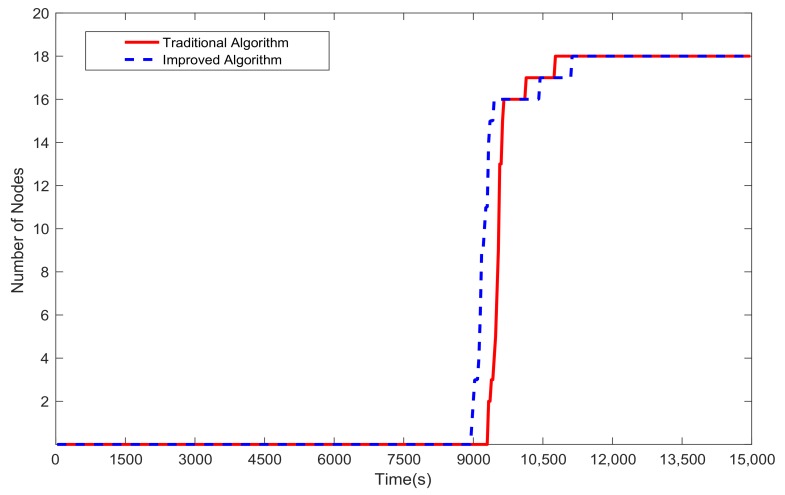
The time-varying number of key nodes with residual energy less than τ in the two algorithms.

Figure 9 shows the time-varying number of key nodes with residual energy less than τ in the two algorithms. Compared with the key nodes with residual energy less than τ in the traditional algorithm (the red solid line), they appear first in the improved algorithm (the blue dashed line) and increase dramatically. After 360 s, key nodes with residual energy less than τ show up in the traditional algorithm when the number of these nodes rises to 14 in the improved algorithm. Therefore, in the late stage of the simulation, for more available key nodes, there is a lower packet loss ratio in the traditional algorithm than in the improved algorithm.

As can be seen in Figure 6a and Figure 9, in the final stage, there are only two malicious nodes (a and b) with residual energy more than τ, and they consume energy slowly. Both algorithms try not to select a malicious node as a relay node, and malicious nodes do not forward all packets even if they are selected as relay nodes. Therefore, the malicious nodes consume less energy than the other 16 nodes.

**Figure 10:**
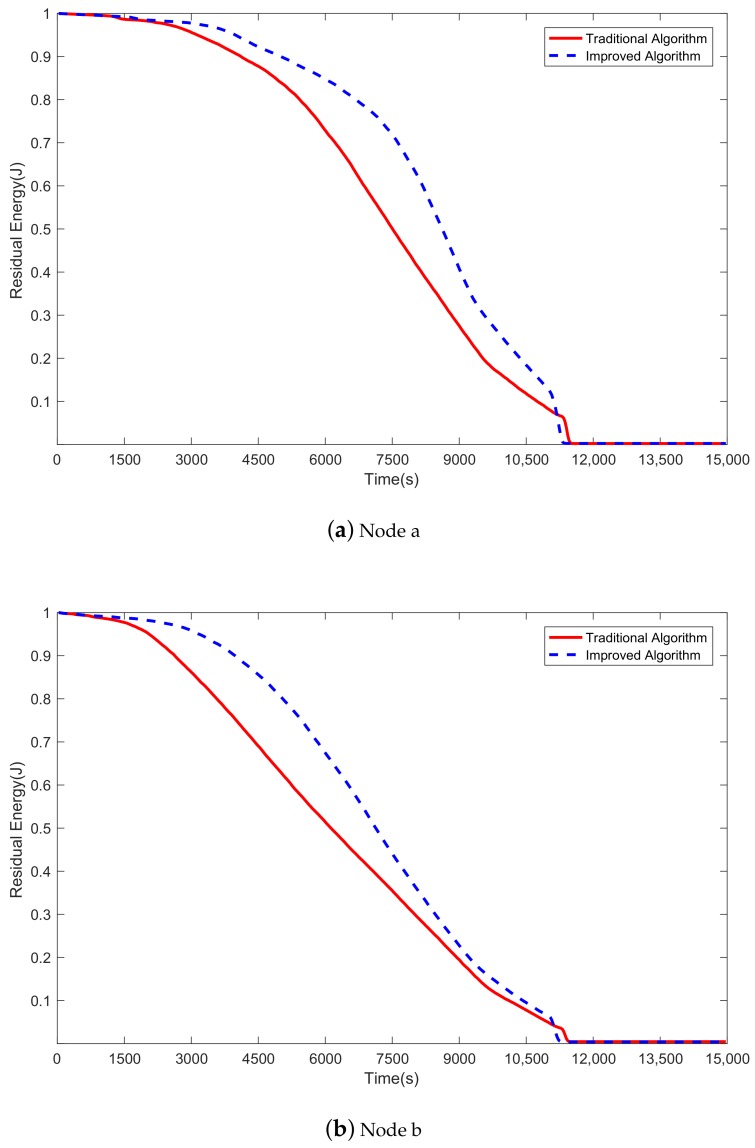
Comparison of energy consumption of malicious nodes a and b between the improved algorithm and the traditional algorithm.

Figure 10a,b show the energy consumption of malicious nodes a and b in the two algorithms, respectively. As can be seen from both figures, in the initial period, the energy consumption of malicious nodes a and b in both algorithms is approximate. After a short period, the energy consumption of the nodes in the improved algorithm (the blue dashed line) is significantly lower than that in the traditional algorithm (the red solid line) because the improved algorithm is superior to the traditional algorithm in terms of avoiding malicious nodes. With a lower probability of malicious nodes being selected as the relay node, less energy is consumed by malicious nodes. Hence, the improved algorithm is better than the traditional algorithm.

### 6.4. G4 Simulations: Dynamic Simulation

**Figure 11:**
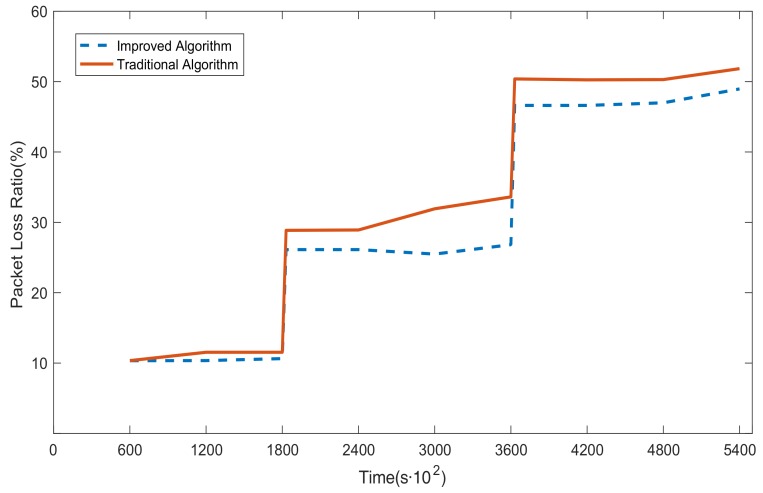
Trend of the packet loss ratio in two algorithms during the dynamic change in the number of malicious nodes.

Figure 11 plots the packet loss ratio in two algorithms when the proportion of the malicious nodes in the WSN increases from 20% to 40% (at 1800 s) and then to 60% (at 3600 s). As we can see from the figure, the packet loss ratio grows significantly when the number of malicious nodes increases. The packet loss ratio gradually converges to a steady value after a short learning time. In Figure 11, the packet loss ratio in the improved Dijkstra algorithm (the blue dashed line) is lower than that in the traditional Dijkstra algorithm (the red solid line) while the number of malicious nodes changes. When the number of malicious nodes that need to be avoided is relatively small, the simulation results show that the packet loss ratio of the improved algorithm is close to that of the traditional algorithm. When the number of malicious nodes approaches half of the total, the improved algorithm obviously outperforms the traditional algorithm. This is due to the fact that the weight calculating method in the improved algorithm is more reasonable and effective than the traditional method, so that the route generated by the improved algorithm can stay away from malicious nodes to the greatest extent possible. Thus, the improved algorithm is superior to the traditional algorithm while the number of malicious nodes changes.

The simulation results verify that the model can learn quickly and maximize network connectivity and the packet delivery ratio, even when the malicious nodes are dynamically increasing. Thus, the intrusion tolerance and robustness of the network can be enhanced.

### 6.5. G5 Simulations: Random Topology Simulation

**Figure 12:**
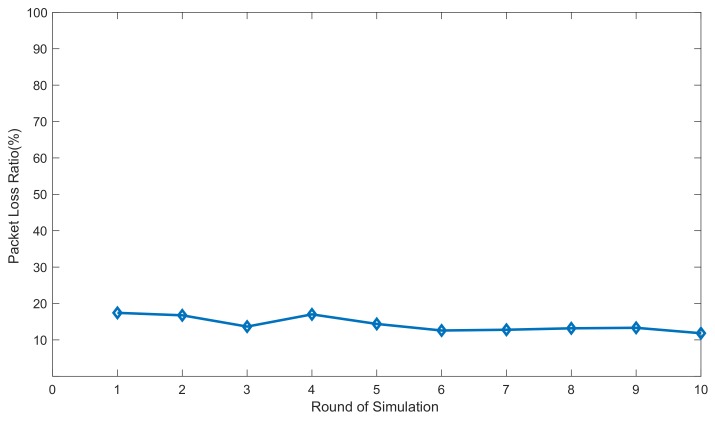
Packet loss ratios in ten independent topologies.

In this group of simulations, to examine the influence of topology on our model, we simulated ten random topologies of the WSN. Figure 12 plots the packet loss ratios for these topologies. As we can see from the figure, the packet loss ratios, which range from 11% to 18%, show little change among the ten different topologies. Therefore, network topology has little influence on the performance of the model, and the model is robust to various WSN topologies.

## 7. Conclusions

In this work, we investigated the secure routing problem for WSNs in the presence of malicious nodes. First, we established a network model for multi-hop communications between sensor nodes and the sink node in the presence of malicious nodes in WSNs. The model describes the behaviors of sensor nodes in WSNs. Second, a method for calculating trust between adjacent nodes was determined as the basis for the route selection. The attack probability of the node is used as the metric for the trust of the node, and it depends on the past behaviors of the node. The attack probability of a path is calculated on the basis of the attack probability of each node in the path and the serial–parallel system in reliability theory. Third, we developed a secure routing model based on information-aware sensor nodes in WSNs. The cost function is based on the node information (attack probability of the K-hop path, the status of each node in the path). The sensor node chooses a path with the least cost required to relay the packet to the sink node. Fourth, an improved version of the Dijkstra algorithm was designed to make it more suitable for our model. Finally, we conducted extensive simulations to verify the validity of our model and algorithm.

In the proposed model, the nodes that are closer to the sink node are chosen as relay nodes to save energy and reduce transmission delay. However, the transmission delay of each path is not specifically quantified, and the upper limit of the delay is not set. In the future, we will consider transmission delay as a factor for path selection to make the secure routing model more realistic.

## Figures and Tables

**Table 1:** Comparison of the improved Dijkstra algorithm and the traditional Dijkstra algorithm.

Algorithm	Weight Factor	Weight Calculation (Node)	Weight Calculation (Path)
Traditional Dijkstra	$Attack_{\langle i,j\rangle }$, $e_{rj}$, $e_{\langle j,sink\rangle }$	$Attack_{\langle i,j\rangle }$+$E_{j}$	Sum of all node weights in a path
Improved Dijkstra	$Attack_{\langle i,j\rangle }$, $e_{rj}$, $e_{\langle j,sink\rangle }$		Equation (9)

**Table 2:** Parameters in the model.

Parameter	Description
$E_{ini}$	Initial energy
M×M	Simulation area
*N*	Number of nodes
SN	Location of sink node
$E_{elec}$	Energy consumption of electronic devices for accepting and transmitting 1-bit data
$\varepsilon_{fs}$	Free space factor
*R*	Transmission radius
η	Adjustment factor
τ	Minimum forwarding threshold

**Table 3:** The setting of parameters.

Parameter	Value
$E_{ini}$	1 J
M×M	100×100 m
*N*	100
SN	50,125
Channel model	Free space
$E_{elec}$	50 nJ/bit
$\varepsilon_{fs}$	10 pJ/bit/m${}^{2}$
*R*	50 m
η	1000
τ	0.1 J
